# Perioperative Care of an Eleven-Year-Old Child With Osteogenesis Imperfecta Type II During Posterior Spinal Fusion

**DOI:** 10.14740/jmc5257

**Published:** 2026-02-02

**Authors:** Alaa Soliman, Marwan Hillis, Allen Kadado, David P. Martin, Joseph D. Tobias

**Affiliations:** a Department of Anesthesiology & Pain Medicine, Nationwide Children’s Hospital, Columbus, OH, USA; b Department of Pediatric Orthopedic Surgery, Nationwide Children’s Hospital, Columbus, OH, USA; c Department of Anesthesiology & Pain Medicine, The Ohio State University College of Medicine, Columbus, OH, USA

**Keywords:** Osteogenesis imperfecta, Pediatric anesthesia, Posterior spinal fusion, Scoliosis

## Abstract

Osteogenesis imperfecta (OI) is an autosomal dominant inherited condition involving connective tissue associated with not only bone fragility, but multi-system involvement with perioperative implications. OI is characterized by bone fragility, skeletal deformities, and variable involvement of the cardiovascular, respiratory, and hematologic systems. Perioperative care poses unique challenges related to the potential for difficult airway management, fracture risk during positioning, restrictive lung disease, coagulation disturbances, and potential hyperthermic reactions. We describe the anesthetic management of an 11-year-old child with OI type II presenting for posterior spinal fusion for progressive scoliosis. The basic cellular mechanisms responsible for OI are reviewed, clinical phenotypic categories and end-organ involvement discussed, and options for intraoperative anesthetic care presented.

## Introduction

Osteogenesis imperfecta (OI), with an estimated incidence of 1 in every 10,000–20,000 live births, is an autosomal dominant disorder resulting from deficient synthesis of type I collagen, a primary component of the extracellular matrix of bone and skin [1–4]. Type I collagen is composed of three amino acid chains that are rich in glycine, proline, and hydroxyproline with glycine occupying every third position along the chain. The high glycine content in these positions is critical for the normal helical formation of the collagen as its small side chain can be incorporated into the spatial constraints of the helix [5]. Although several sub-types have been described, in the majority of cases, the gene defect results in the substitution of a different amino acid instead of glycine thereby disrupting the collagen helix. This defect leads to the classic triad of osteogenesis imperfecta including susceptibility to fractures, blue sclerae, and conductive hearing loss. Spinal involvement may include scoliosis, kyphosis, cranio-cervical junction abnormalities, and lumbosacral pathology [6, 7].

Perioperative management in OI is challenging because of bone fragility, fracture risk, difficulties with airway management, impaired pulmonary function with restrictive pulmonary mechanics, bleeding diathesis related to platelet dysfunction, and temperature dysregulation manifesting as intraoperative hyperthermia or hypothermia [8, 9]. We present an 11-year-old child with OI type II who presented for anesthetic care during posterior spinal fusion. We explore the genetic history and diversity of this disorder, outline end-organ involvement, and discuss perioperative concerns.

## Case Report

Review of this case and presentation in this format followed the guidelines of the Institutional Review Board of Nationwide Children’s Hospital. This review was conducted in compliance with the ethical standards of the responsible institution on human subjects as well as with the Helsinki Declaration.

**Figure 1:**
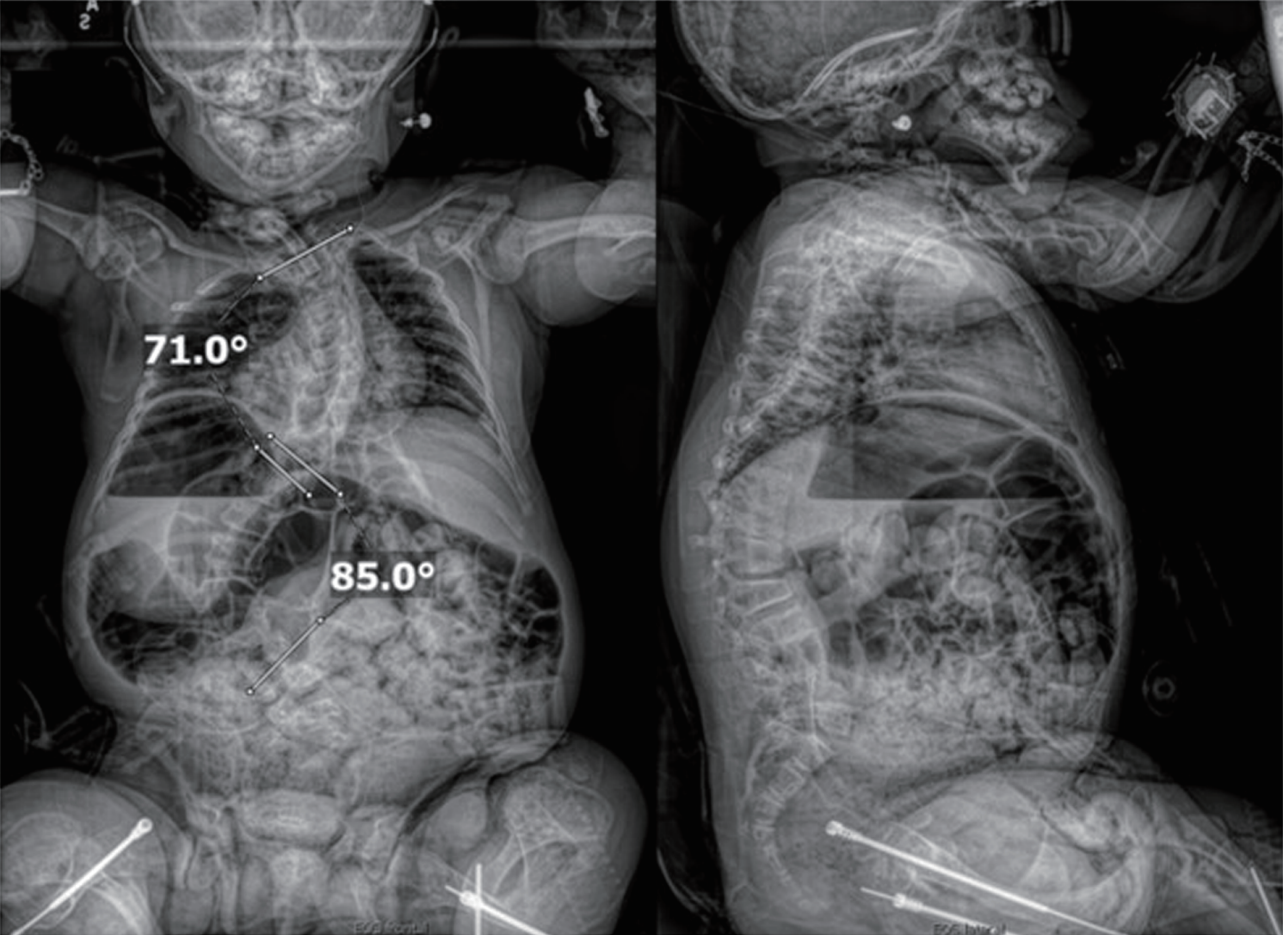
Preoperative seated radiographs demonstrating severe scoliosis with 71° right thoracic curve (T1-T9), 85° left lumbar curve (T9-L3, major), and thoracolumbar kyphosis.

**Figure 2:**
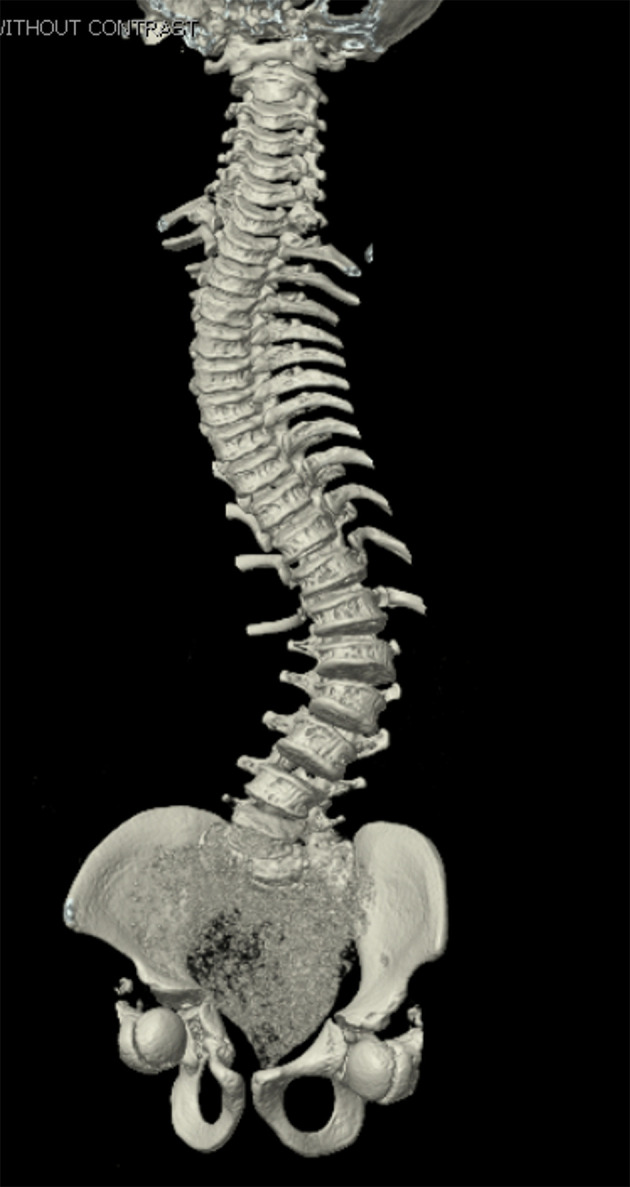
Preoperative computed tomography reconstruction of the vertebral column showing severe scoliosis.

**Figure 3:**
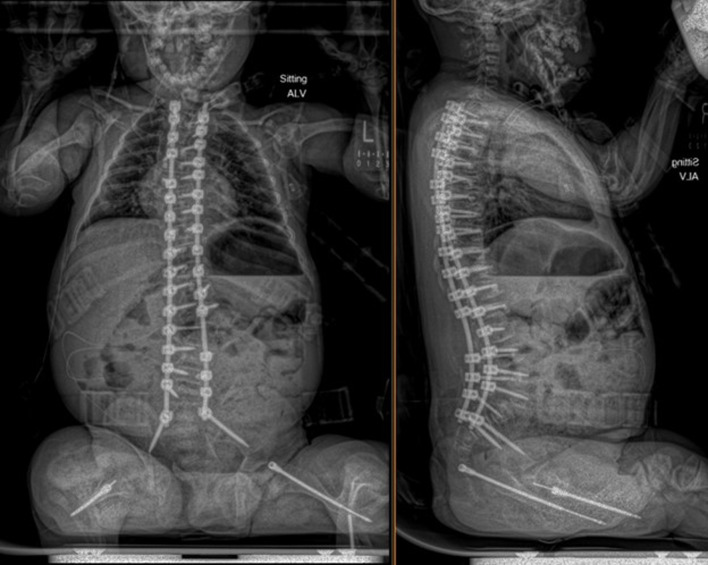
Postoperative seated radiographs following scoliosis correction with T2 to pelvis posterior spinal instrumented fusion, posterior column osteotomies, and cement vertebral screw augmentation.

An 11-year-old male, prepubertal child with a diagnosis of OI type II and severe scoliosis presented for T2-pelvis posterior spinal instrumented fusion with posterior column osteotomies and cement vertebral screw augmentation. Preoperative evaluation revealed a 71° thoracic curve and an 85° lumbar curve (Figs 1 and 2). Despite the rarity of survival of a patient with OI type II to this age, the diagnosis was based on DNA testing which documented that the patient was heterozygous for a COL1A1 pathogenic mutation. This mutation converts a highly conserved glycine at amino acid #302 to valine. These mutations have been seen in OI types 1–4. Based on this and the clinical findings at birth, the medical geneticist specialist opined that the patient’s presentation was consistent with OI type II and that no further genetic evaluation was needed. Additional complex past medical history included Chiari I malformation, hydrocephalus status post ventriculoperitoneal (VP) shunt, restrictive lung disease, and chronic tracheostomy with ventilator dependence. The patient also had bowel and bladder incontinence, and a history of multiple long-bone fractures requiring prior orthopedic interventions, including bilateral tibial and femoral osteotomies. Growth parameters were significantly low with a weight of 15.8 kg and height of 90 cm, both below the first percentile for age. The diagnosis of OI was first suspected prenatally when a 20-week ultrasound revealed skeletal anomalies. Delivery was at term via scheduled cesarean section to a 30-year-old mother (G2P1). At birth, the neonate required oxygen supplementation and stimulation for resuscitation, with Apgar scores of 1, 4, and 8 at 1, 5, and 10 min, respectively. Early postnatal imaging showed multiple rib fractures, bowing of long bones, and deformities of the clavicle and femur, consistent with severe OI. Genetic analysis subsequently confirmed the diagnosis of OI type II, identifying a heterozygous COL1A1 pathogenic mutation (c.905G>T) resulting in substitution of glycine with valine at position 302—a mutation known to cause OI types I–IV. Following birth, the infant exhibited respiratory failure due to pulmonary hypoplasia and multiple rib fractures, leading to early ventilator dependence. At 4 months of age, a tracheostomy was performed due to progressive and continued respiratory failure and chronic ventilator dependence. Subsequently, hydrocephalus and Chiari I malformation were identified on magnetic resonance imaging that required placement of a VP shunt. The past surgical history revealed multiple surgical interventions to treat the multisystem effects of OI. These included placement of a feeding gastrostomy, circumcision, tracheostomy, and VP shunt. Orthopedic management involved bilateral tibial osteoplasties with intramedullary rodding and a left tibia and fibula osteotomy with fixation. All previous surgeries were performed under general anesthesia without complications. Current medications included nebulized albuterol every 6 h as needed; cetirizine 10 mg once a day as needed for allergy symptoms; nebulized sodium chloride 0.9% every 4 h as needed for secretion management; calcium carbonate suspension (500 mg (twice a day). Additionally, to promote bone health, a bisphosphonate infusion (zoledronic acid 0.05 mg/kg) was provided every 12 weeks. The bowel regimen included polyethylene oral powder via the gastrostomy tube, enteral sennosides, and glycerin or bisacodyl suppositories as needed. Management of chronic pain included alternating doses of acetaminophen and ibuprofen as needed. Current allergies included pork-derived products, adhesive tape, and environmental allergens. Nutrition was maintained with Boost Kid Essentials oral supplement. Pre-anesthetic physical examination revealed a young child who was tracheostomy and ventilator dependent. Ventilator support was provided by a Trilogy home ventilator. Settings include peak inspiratory pressure 24 cm H_2_O, positive end expiratory pressure (PEEP) 8 cm H_2_O, pressure support 16 cm H_2_O, rate 10 breaths/min with oxygen 0.5–1 L/min as needed to maintain the oxygen saturation. Breath sounds were clear. The cardiovascular examination was unremarkable with a heart rate of 100–110 beats/min. A blood pressure was not obtained due to bone fragility concerns. Preoperative complete blood count and coagulation profile were within normal limits. Additional preoperative laboratory values revealed serum calcium 7.4 mg/dL (normal 8–10 mg/dL), phosphorous 3.8 mg/dL (normal 3.7–5.6 mg/dL), and alkaline phosphatase 115 U/L (normal 137–424 U/L). Preoperative echocardiogram revealed mild pulmonary regurgitation and a patent foramen ovale. Given his severe OI and associated comorbidities, anesthetic planning emphasized fracture prevention during positioning, maintenance of normothermia, careful airway and ventilatory management through the tracheostomy, and readiness for significant blood loss. The patient was assigned an American Society of Anesthesiologists’ (ASA) physical classification IV due to severe systemic disease and ventilator dependence. Multidisciplinary coordination between anesthesiology, orthopedic surgery, pulmonology, and critical care teams was arranged, with plans for postoperative pediatric intensive care unit (PICU) admission for ventilatory and hemodynamic support and monitoring. Pre-medication included aprepitant to prevent postoperative nausea and vomiting as well as gabapentin as part of our usual postoperative pain management regimen. The patient was held nil per os after midnight and transported to the operating room where routine ASA monitors were placed. Anesthesia was induced by the inhalation of incremental concentrations sevoflurane in 50% oxygen and nitrous oxide through the existing tracheostomy. After the induction of anesthesia, two peripheral intravenous cannulas were placed (18 and 18 gauge) with a loosely placed tourniquet to facility identification of the veins. A left ulnar arterial cannula was placed with ultrasound guidance following a negative Allen’s test. A urinary catheter with a core temperature probe was inserted. Maintenance anesthesia included our routine practice to facilitate intraoperative neurophysiologic monitoring. This included total intravenous anesthesia with propofol (50–150 µg/kg/min) and remimazolam (5–10 µg/kg/min) titrated to maintain the bispectral index at 50–60, methadone (0.15 mg/kg) and remifentanil (0.1–0.3 µg/kg/min) adjusted according to the mean arterial pressure (MAP), and lidocaine (1 mg/kg/h). Intraoperative mechanical ventilation was provided by a pressure-regulated, volume-guaranteed mode with a tidal volume of 8 mL/kg and PEEP 5 cm H_2_O. The rate was adjusted to maintain the ETCO_2_ between 35 and 40 mm Hg and the FiO_2_ was adjusted to maintain the oxygen saturation greater than 95%. Prophylaxis against surgical site infection included cefazolin 50 mg/kg every 3 h and a single dose of gentamicin (5 mg/kg). Limitation of the needed for allogeneic transfusions included maintenance of the MAP at 55–65 mm Hg, intraoperative cell saver, a single dose of desmopressin (0.15 units/kg), desmopressin (0.15 µg/kg), and tranexamic acid infusion (50 mg/kg followed by an intraoperative infusion at 5 mg/kg/h). After instrumentation, the MAP was increased as needed by a phenylephrine infusion (0.5–1 µg/kg/min). Intraoperative temperature was managed using a forced air warming device. Prone positioning was performed cautiously and carefully, with meticulous attention to protecting pressure points, securing the airway and maintaining spinal alignment throughout positioning. The patient’s existing right frontal VP shunt was identified and protected throughout positioning. Intraoperative fluids included Normosol-R (850 mL), 5% albumin (250 mL), and a unit of packed red blood cells (320 mL). Urine output was 520 mL with an estimated blood loss of 600 mL. The surgical procedure included posterior spinal fusion with instrumentation from T2 to the pelvis with cemented vertebral screw augmentation (n = 22) from T2–L1 on the right and at T2, T4, T5, T7–11, and L3–4 on the left with posterior column osteotomies from L11–T2. After completion of the 11-h surgical procedure, the patient was turned supine, and ventilation was maintained through the tracheostomy with a patent, unobstructed airway. The cardiovascular status remained stable. The patient was awake, responsive, and followed commands. There were no complaints of pain or nausea. The patient was transported directly from the operating room to the PICU with the anesthesia team in attendance. Postoperative pain was managed with intermittent acetaminophen, a lidocaine infusion, and morphine delivered by nurse-controlled analgesia, which was subsequently transitioned to oral acetaminophen, ibuprofen, valium, and oxycodone managed under the guidance of the pain service. Physical and occupational therapy were involved throughout the hospital stay. Postoperative imaging showed well-aligned spinal instrumentation without concerns (Fig. 3). Routine home medications were restarted. The remainder of the postoperative course was uneventful, and he was discharged home on postoperative day 5. Follow-up at 3 months postoperatively with radiographic imaging demonstrated overall appropriate alignment and positioning of all hardware.

## Discussion

In 1979, Sillence proposed a classification of OI into types I, II, III, and IV, which was based on clinical findings with a radiological and mode of inheritance sub-classification of OI type II into A, B, C [1]. In 2004, Rauch and Glorieux published an extension of the original Sillence classification, introducing OI types V–VII to include phenotypically distinct forms of the disease, based on clinical and inheritance patterns [10]. OI type V was described as a moderate form with acknowledged autosomal dominant inheritance, whereas types VI and VII were identified as autosomal recessive variants, each associated with unique histopathologic and radiographic characteristics. Sillence has further classified OI type II into subtypes IIA, IIB, and IIC based on evident radiologic features [11, 12]. Forlino and Marini in 2016, proposed a functional metabolic classification that groups OI into five categories according to the affected molecular pathway: (A) defects in collagen synthesis, structure, or processing (COL1A1, COL1A2, BMP1); (B) collagen modification defects (CRTAP, LEPRE1, PPIB, TMEM38B); (C) collagen folding and cross-linking defects (SERPINH1, FKBP10, PLOD2); (D) defects in ossification and mineralization (IFITM5, SERPINF1); and (E) defects in osteoblast differentiation and collagen insufficiency (WNT1, CREB3L1, SP7) [6]. The first autosomal recessively inherited case of OI type II was found to be related to mutations in the CRTAP gene in 2006 [13]. The OI classification system has gradually expanded to include up to 14 different types based on underlying molecular abnormalities because of later discoveries of additional gene defects [14].

OI type II, the disorder found in our patient, is typically a perinatally lethal form of the disorder, classically resulting from dominant de novo mutations in the COL1A1 or COL1A2 genes, which encode the proα1(I) and proα2(I) chains of type I collagen [1, 6]. These mutations disrupt the formation and stability of the collagen triple helix, leading to severe defects in collagen synthesis, extreme bone fragility, and intrauterine fractures [15, 16]. Clinically, OI type II presents with multiple fractures at birth, shortened and bowed bones, blue sclerae, a small thoracic cage, and pulmonary hypoplasia, often leading to respiratory failure shortly after delivery [11–13].

Skeletal involvement in this subtype represents the most severe and usually fatal form of the disease in perinatal life with the diffuse skeletal fragility and deformity affecting almost all bones. The long bones, particularly the femur, tibia, and humerus, are compressed, appearing short, broad, and bowed with a specific appearance on radiographs. Multiple intrauterine fractures are also commonly seen [12]. Reduced bone mineralization, particularly manifest in the pelvis and scapulae, further compromises skeletal stability. The ribs are thin and frequently fractured. Progressive deformities from kyphoscoliosis and multiple vertebral compression fractures result in thoracic and chest wall deformity impairing pulmonary development [10, 17]. These complications led to respiratory failure during the neonatal period in our patient from restrictive lung disease necessitating tracheostomy and chronic mechanical ventilation.

Cranial and cervical vertebral involvement results in basilar invagination, reduced ossification, and cranio-vertebral concerns including atlanto-axial subluxation [12, 13]. Neurovascular involvement of the CNS includes cavernous fistulas of the carotid artery, carotid artery dissection, and cerebral aneurysms. The brain parenchyma may be affected resulting in cerebral atrophy, communicating hydrocephalus, and cerebellar hypoplasia [18, 19].

Collagen involvement may also affect the heart and great vessels resulting in valvular insufficiency (most commonly mitral and aortic regurgitation) and aortic dilatation [20–22]. The magnitude of involvement generally results in only mild regurgitant lesions. More significant involvement may rarely result in progressive aortic root dilation, and aortic dissection and coronary artery dissection have also been reported rarely [21]. Defects in collagen synthesis may lead to altered ventricular compliance and impaired systolic function. There is also a report associated with congenital heart disease (CHD) including patent ductus arteriosus (PDA) and atrial septal defect (ASD) [20]. Given these concerns, preoperative echocardiography is recommended for all patients. Guidelines provided by the American Heart Association should be followed in patients with valvular dysfunction or CHD as they may require prophylactic antibiotics to prevent subacute bacterial endocarditis based on the type of surgical procedure.

Coagulation disturbances in patients with OI may result from capillary and connective tissue fragility along with abnormal platelet function manifested as diminished aggregation responses and prolonged bleeding times [23]. In some cases, von Willebrand–like abnormalities have also been observed. This combination of vascular and platelet dysfunction, together with the extensive dissection required during orthopedic surgery may increase intraoperative blood loss. Consequently, the preoperative evaluation should include a complete blood count, evaluation of routine coagulation studies (prothrombin time (PT), partial thromboplastin time (PTT), and international normalized ratio (INR)), as well as a more comprehensive evaluation of platelet function based on the patient’s clinical history [24]. Intraoperative management should include availability of blood products, adequate vascular access for intraoperative resuscitation, and pharmacologic augmentation of coagulation function including use of anti-fibrinolytic agents (tranexamic acid) and consideration for administration of additional agents to augment platelet function (desmopressin), which ensure hemostatic stability and emphasize gentle tissue handling [8, 25].

Published experience for perioperative care of patients with type II OI is limited. We were able to identify only two previous reports of anesthetic care in patients with OI undergoing spinal fusion surgery (Table 1) [26, 27]. These cases and the one we report outline the important considerations in this patient including attention to airway management and potential difficulties related involvement of the cranio-cervical junction, intraoperative positioning and fracture risk including pressure from non-invasive blood pressure (NIBP) devices, obtainment of arterial access, and intraoperative blood and fluid administration with use of techniques to avoid or limit the need for allogeneic blood transfusion (tranexamic acid, desmopressin, intraoperative cell salvage) [28, 29]. These and other perioperative concerns are outlined in Table 2.

**Table 1:** Previous Reports of Anesthetic Care in Patients With Osteogenesis Imperfecta Undergoing Posterior Spinal Fusion

Author and reference	Patient demographic	Anesthetic technique	Comments and outcome
Patino and Soliman [26]	A 4-year-old, 11 kg girl with OI and severe scoliosis (Cobb angle 90°) for PSF (T2–L3). Prior history of multiple atraumatic fractures and restrictive lung disease.	Induction and maintenance: Intravenous induction with propofol, oxygen/nitrous oxide, isoflurane, and remifentanil (0.1–0.3 µg/kg/min). Airway approach: Gentle mask ventilation and direct laryngoscopy with neutral head positioning.	Attention to prone positioning with pressure points padded. Invasive BP monitoring and avoidance of noninvasive blood pressure monitoring due to fracture risk. Cell saver to limit need for allogeneic blood. Patient’s trachea was extubated uneventfully, transferred to the PICU with no new fractures or complications. Uncomplicated postoperative course.
Chin and Stuart [27]	An 11-year-old, 21.6 kg boy with autosomal recessive OI type VIII (LEPRE1 mutation) for posterior spinal fusion (T3–L5) for progressive scoliosis (Cobb angle 54.5°). Prior history of extreme bone fragility, barrel chest, visual/hearing impairment, and restricted growth.	Induction and maintenance: Total intravenous anesthesia (TIVA) using target-controlled propofol and remifentanil. Airway approach: Endotracheal intubation by direct laryngoscopy with minimal neck movement and head in neutral position.	Invasive BP monitoring. Blood conservation strategies included intraoperative cell salvage and tranexamic acid. Temperature management included forced air warming and in-line fluid warmer. The patient’s trachea was extubated on postoperative day 1 but later sustained humeral and rib fractures (conservative management).

BP: blood pressure; OI: osteogenesis imperfecta; PSF: posterior spinal fusion.

**Table 2:** Perioperative Concerns in Patients With Osteogenesis Imperfecta

1. Airway concerns Atlanto-occipital subluxation (potential for spinal cord injury during airway manipulation) Abnormal dentition – risk of dental trauma Difficult bag-valve-mask ventilation and endotracheal intubation (short neck, dental problems, micrognathia, midface and mandibular deformities, limited neck extension) Tracheostomy
2. Progressive kyphoscoliosis and thoracic wall deformities Respiratory insufficiency (restrictive lung disease, decreased pulmonary compliance) Pulmonary hypoplasia can cause chronic hypoxemia and postoperative respiratory failure Postoperative ventilatory support
3. Cardiac involvement Valvular insufficiency (aortic and mitral regurgitation) Heart failure due to collagen defects affecting myocardium Progressive aortic dilatation or rarely dissection Associated congenital heart disease
4. Bleeding tending Capillary fragility Abnormal platelet aggregation with prolonged bleeding time Pharmacologic manipulation of coagulation cascade and platelet function (antifibrinolytic therapy and DDAVP) Availability of blood and blood products
5. Bone fragility Fractures may occur during transfer, positioning, or NIBP cuff use Attention to positioning and padding Avoid excessive manipulation of limbs or neck Limb deformities and previous fractures may make IV and arterial access difficult NIBP cuffs, if used, should be placed over well-padded areas and cycled minimally
6. Difficult venous and arterial access Tourniquet to be loosely placed for IV access Consider invasive arterial monitoring Ultrasound-guided techniques may be preferred
7. Positioning concerns Use soft padding under pressure points Neutral head positioning and minimal neck movement Head rings or foam supports to prevent cervical injury Transfers and repositioning should involve multiple staff members
8. Potential for intraoperative hyperthermia Not malignant hyperthermia related Continuous temperature monitoring Maintain normothermia using warming/cooling devices as needed
9. Central nervous system involvement Chiari malformation commonly occurs in OI type II (potential brainstem compression and cervical instability) with potential for cranial nerve involvement Cranio-vertebral junction involvement with atlanto-axial subluxation and potential for cervical spinal cord injury Hydrocephalus

IV: intravenous; NOBP: non-invasive blood pressure; OI: osteogenesis imperfecta.

### Learning points

In summary, we present the successful anesthetic management of an 11-year-old child diagnosed with OI type II, who presented for posterior spinal fusion for scoliosis. This case illustrates the essential role of preoperative planning, thoughtful intraoperative management, and vigilant postoperative care in OI patients. The perioperative care of these patients begins with a comprehensive evaluation of the patient’s past medical history, their current clinical status, specific end-organ involvement of OI, and the intended surgical procedure. Of primary importance to perioperative care is airway management with the potential for difficulties with bag-valve-mask ventilation and endotracheal intubation. Neuromuscular blocking agents (NMBAs) should not be administered until effective bag-valve-mask ventilation is demonstrated and the equipment for dealing with the difficult airway including indirect videolaryngoscopy should be available. Additional challenges include a bone fragility and extremity deformity mandating care regarding positioning, vascular access, and patient movement. Restrictive lung disease and pulmonary hypoplasia mandate tailored ventilatory strategies and planned postoperative respiratory support. Coagulation disturbances include platelet dysfunction which may require blood conservation strategies including antifibrinolytic agents, DDAVP, intraoperative cell salvage, and ensuring the availability of blood products with adequate vascular access for transfusion. Preoperative echocardiography may be indicated to identify associated valvular dysfunction or aortic root dilatation. The success observed in this case demonstrates the benefits of a tailored anesthetic approach for patients with OI type II undergoing orthopedic surgery with a multidisciplinary approach and a collaborative effort of anesthesiologists, orthopedic surgeons, and other healthcare professionals.

## Data Availability

Any inquiries regarding supporting data availability of this study should be directed to the corresponding author.
